# Supplementary material for: Topologically protected entanglement switching around exceptional points
Source: Light Sci Appl. 2024 Jul 16;13:167. doi: 10.1038/s41377-024-01514-1 (PMC11252316; doi:10.1038/s41377-024-01514-1)
Supplement: Supplementary file 1 — SP [file 41377_2024_1514_MOESM1_ESM.pdf]

Supplementary Information for "Topologically protected entanglement switching around exceptional points"

Zan Tang,* Tian Chen,*+ Xing Tang, and Xiangdong Zhang$

Key Laboratory of Advanced Optoelectronic Quantum Architecture and Measurements of Ministry of Education, Beijing Key Laboratory of Nanophotonics & Ultrafine Optoelectronic Systems, School of Physics, Beijing Institute of Technology, 100081, Beijing, China

*These authors contributed equally to this work. $+To whom correspondence should be addressed. E-mail: zhangxd@bit.edu.cn; chentian@bit.edu.cn

S1. Theoretical results of each step evolution along Loop 1.

The theoretical results for encircling the EP along Loop 1 have been provided in Fig. S1. We assume that after n-steps evolution, the input state changes to state $\left|X_n\right\rangle$, and this state can be decomposed as $\left|X_n\right\rangle = C_1\left|\alpha_{G1}\right\rangle + C_2\left|\alpha_{L1}\right\rangle + C_3\left|\alpha_{G2}\right\rangle + C_4\left|\alpha_{L2}\right\rangle$, where $\left|\alpha_{G1}\right\rangle$ and $\left|\alpha_{G2}\right\rangle$ are the instantaneous gain eigenstates with eigenvalue imaginary part $\mathrm{Im}(\lambda) > 0$, and $\left|\alpha_{L1}\right\rangle$ and $\left|\alpha_{L2}\right\rangle$ are the instantaneous loss eigenstates with eigenvalue imaginary part $\mathrm{Im}(\lambda) < 0$. The amplitudes $\left|C_{1,2,3,4}\right|$ of four instantaneous eigenstates are provided in Fig. S1. For the study of encircling the EP clockwise (Fig. S1a-S1d), when starting from $\left|\zeta_1\right\rangle$ or $\left|\zeta_4\right\rangle$, the state experiences the evolution path on the red Riemann surfaces, i.e. experiencing the gain mode where the imaginary part of the quasienergy is positive. In this case, the input states adiabatically evolve on the Riemann surface, and change to different output states after one cycle of parameter changes, as shown in Figs. S1a and S1d. As a comparison, when the input states are $\left|\zeta_2\right\rangle$ and $\left|\zeta_3\right\rangle$ (Figs. S1b and S1c), the evolution paths at the initial stage are on the blue Riemann surfaces, i.e. the loss mode where the imaginary part of the quasienergy is negative. In this case, the tiny non-adiabatic coupling between the loss and gain modes of the non-Hermitian system induces non-adiabatic transitions (NAT), which breaks the adiabaticity. It results in the transition from the blue Riemann surface to the red one during the evolution.

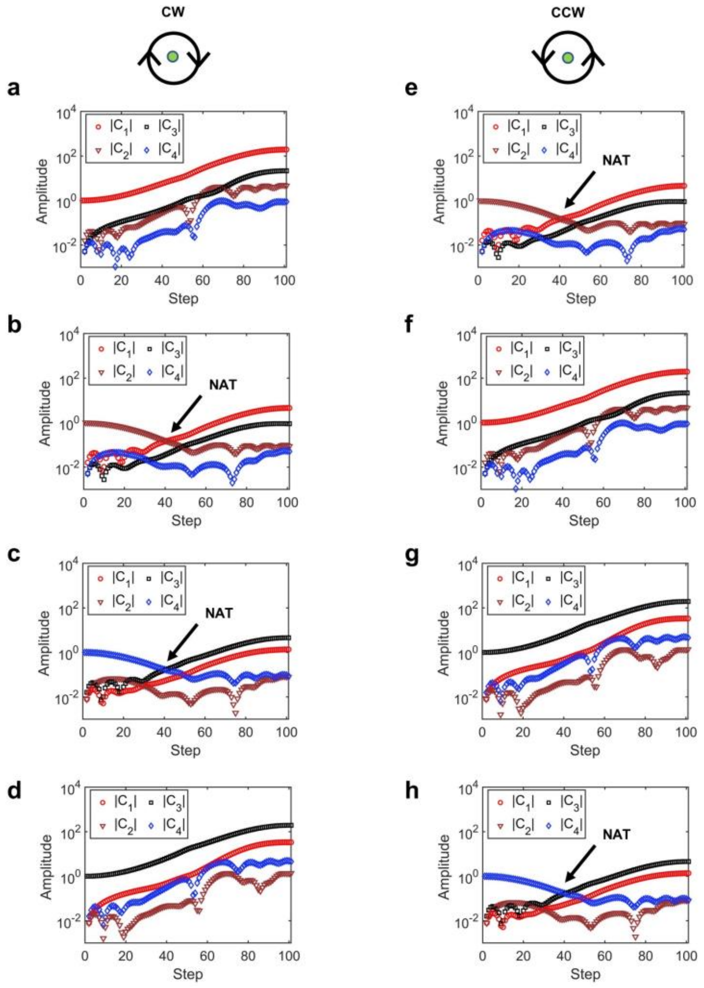

Figure S1. Theoretic results for encircling the EP along Loop 1. a-d the amplitudes of four instantaneous eigenstates during the clockwise (CW) evolution. From top to bottom, the input states are Bell states $|\zeta_1\rangle$ to $|\zeta_4\rangle$. **e-h** the amplitudes of four instantaneous eigenstates during the counter-clockwise (CCW) evolution. From top to bottom, the input states are Bell states $|\zeta_1\rangle$ to $|\zeta_4\rangle$. For all cases, the number of total steps is taken as N=100.

For counter-clockwise evolution, the results in Figs. S1e-S1h are different from the clockwise encircling. Starting from $|\zeta_2\rangle$ or $|\zeta_3\rangle$, the input state experiences the evolution path which is composed by the gain mode. Therefore, these input states evolve adiabatically on the Riemann sheet, and no NAT appears, see Fig. S1f and S1g. When the input state $|\zeta_1\rangle$ or $|\zeta_4\rangle$, the initial stage of the evolution paths are composed of the loss modes. So

the NAT occurs during the evolution, causing the states to return to themselves after one loop, as shown in Figs. S1e and S1h.

S2. Theoretical results of each step evolution along Loop 2.

As mentioned in the main text, the chiral switch disappears if the evolution path of parameters is far away from the EP. To illustrate this phenomenon clearly, we provide the evolution results along a loop far away from the EP in Fig. S2. The parameter values at the *n*th step are chosen as: $\varphi = 0.1\times\cos(\pm\frac{2\pi}{N}n-\frac{\pi}{2})$ and $\theta_1 = 0.1\times\sin(\pm\frac{2\pi}{N}n-\frac{\pi}{2})-0.5$, and it forms a loop not enclosing the EP but away from the EP. This loop is shown by the red Loop 2 in Fig. 1c of the main text.

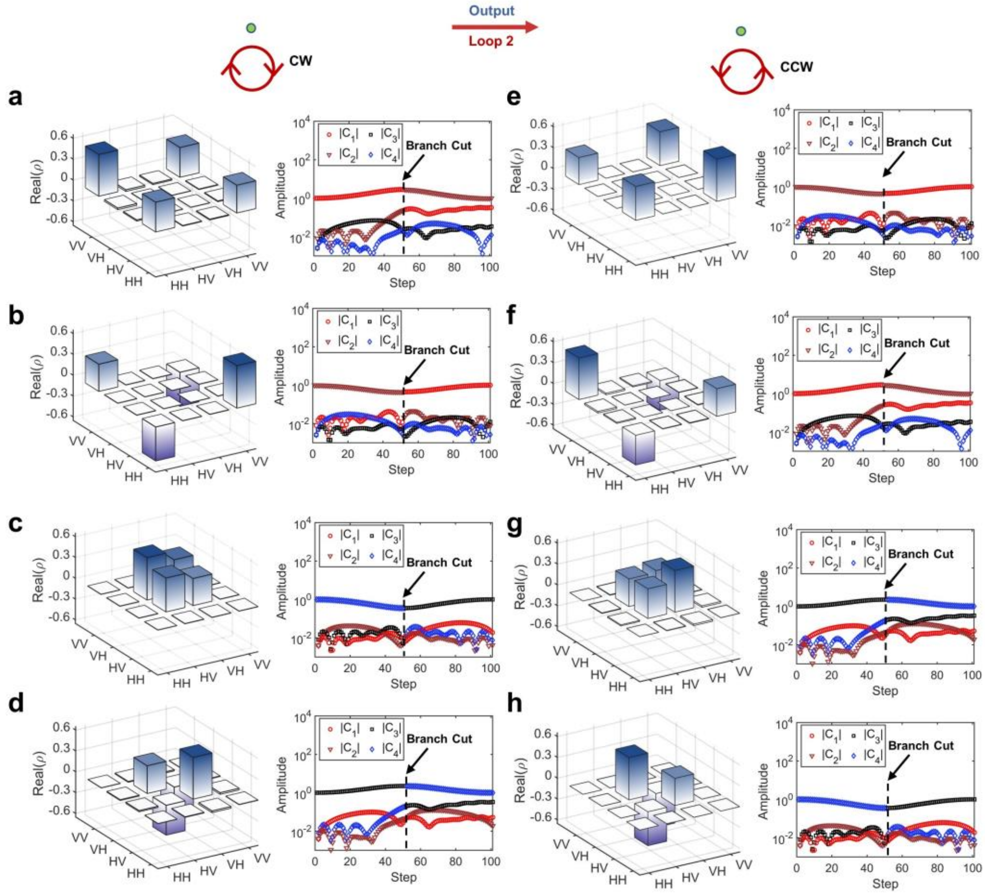

Figure S2. Theoretical results along Loop 2. a-d Density matrices of the output states and the amplitudes of four instantaneous eigenstates during the clockwise evolution. From top to bottom, the input states are Bell states $|\zeta_1\rangle$ to $|\zeta_4\rangle$. **e-h** Density matrices of the output states

and the amplitudes of four instantaneous eigenstates during the couter-clockwise evolution. From top to bottom, the input states are Bell states $|\zeta_1\rangle$ to $|\zeta_4\rangle$. For all cases, the number of total steps is N=100.

Since the parameters in Loop 2 are far away from the EP, the NAT does not appear during the evolution, and only adiabatic evolution can be found for all four input states. The initial states evolve adiabatically along the Riemann energy surfaces, travel across the branch cut and return back to the initial cases. As shown in Fig. S2, no matter whether the initial states evolve clockwise or counter-clockwise, they return back to themselves after one period of parameters. These results indicate that the initial states are not affected by the EP during the evolution, and no chiral switch can be found.

S3. The results with different points in the loop

In the experimental design of the main text, we design the evolution encircling the EP by multi-steps quantum walk (QW) $I \otimes M_{\mathrm{n}}$, which can be experimentally realized. To verify the success of this experimental design, we give the theoretical results with the total number of step $N = 100$. The points in this case are selected equally, see Fig. S3a. As shown by blue bars in Fig. S3d-S3e, it is clearly seen that the output states of this design are nearly the same to those shown in Fig. 2 of the main text. The fidelities between each group of output states are larger than 0.99. Therefore, our experiment design with multi-steps QW is appropriate for the chiral switch of Bell states.

However, due to loss in the experiment, it is impossible to realize a 100-steps QW. Through our calculation, it is found that following the circular trajectory of Loop 1, and the parameters θ_1 and φ are unequally spaced with the total number of steps, the calculated output state is close to the output state for the aforementioned $N = 100$ case. When $N = 16$ (the points are chosen as shown in Fig. S3b), regardless of clockwise or counterclockwise direction, the fidelity remains greater than 0.94 for different input states, as shown by the gray bars in Fig.

S3d and S3e. Even with $N=8$ (the points chosen as shown in Fig. S3c), the fidelity remains greater than 0.85, see the pink bars in Fig. S3d and S3e.

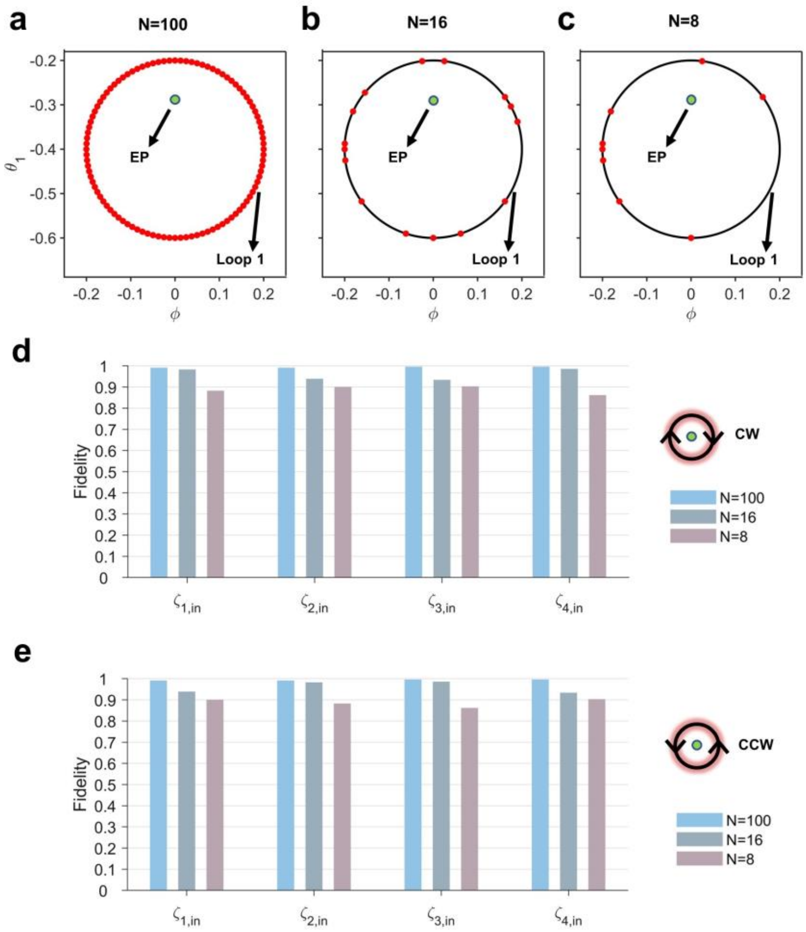

Figure S3. a-c Sampling points along the evolutionary trajectory Loop 1 for different total numbers of steps *N*, where **c** shows the data points used in the experiment. **d** Output states results with different input entangled states during clockwise encircling. **e** Output states results for counter-clockwise encircling. The horizontal axis $|\zeta_{j,in}\rangle (j=1,2,3,4)$ labels the four different input Bell states. The vertical axis represents the fidelity between the output state and the ideal output entangled state.

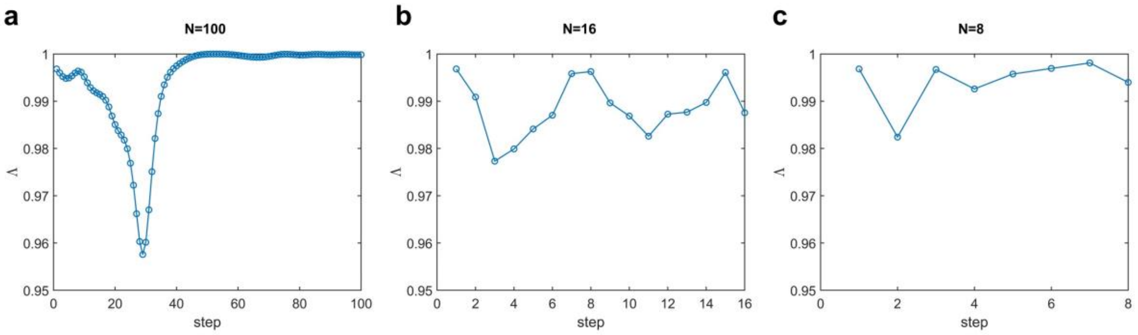

Figure S4. The calculation of the inner product $\Lambda = \left|\left\langle \zeta_{in}^{n} \middle| \zeta_{out}^{n} \right\rangle\right|$ between the output state and input state for each step evolution operator. For **a-c,** the points along Loop 1 are chosen as those in Fig. S3a-S3c**.**

To explore the different choices of points along Loop 1, we calculate the inner product $\Lambda = \left|\left\langle \zeta_{in}^{n} \middle| \zeta_{out}^{n} \right\rangle\right|$ between the output state and input state for each step evolution operator, where the initial state is $\left|\zeta_1\right\rangle$. A smaller Λ indicates a larger change of the output state compared to the input state during each step of evolution. It attributes to the more obvious change of eigenstates of the system when compared with the input state. Figure S4 displays the value Λ with encircling the EP counter-clockwise. It is clearly seen that during the intermediate steps of evolution, these values Λ change obviously. Compared with other steps, the loop in the intermediate steps is closer to the EP, so this change illustrates the fast change of eigenstates near the EP. Moreover, an interesting phenomenon appears in Fig. S4 that when $N = 100$ (Fig. S4a), more values of Λ are smaller than those for $N = 16$ and $N = 8$. So the change of eigenstates affects the evolution less for $N = 16$ (Fig. S4b) and least for $N = 8$ (Fig. S4c). However, it is noted that a bad effect emerges gradually for the cases with the smaller number of points. That is, the evolution with smaller points cannot be a fully adiabatic evolution along the loop. Such evolution can only basically meet the adiabatic condition. Fortunately, the fidelities of output states shown in Fig. S3 indicate that even with $N = 8$, the adiabatic condition is also basically satisfied and the chiral entanglement switch is still apparent.

Considering the feasible realization in the experiment, the points along the loop are chosen as those in Fig. S3c. These 8 points correspond to $\frac{n}{N} = \frac{0}{100}, \frac{35}{100}, \frac{48}{100}, \frac{68}{100}, \frac{74}{100}, \frac{75}{100}, \frac{77}{100}$ and

$\frac{85}{100}$ for the parameters $\varphi = 0.2 \times \cos(\pm 2\pi \frac{n}{N} - \frac{\pi}{2})$ and $\theta_1 = 0.2 \times \sin(\pm 2\pi \frac{n}{N} - \frac{\pi}{2}) - 0.4$.

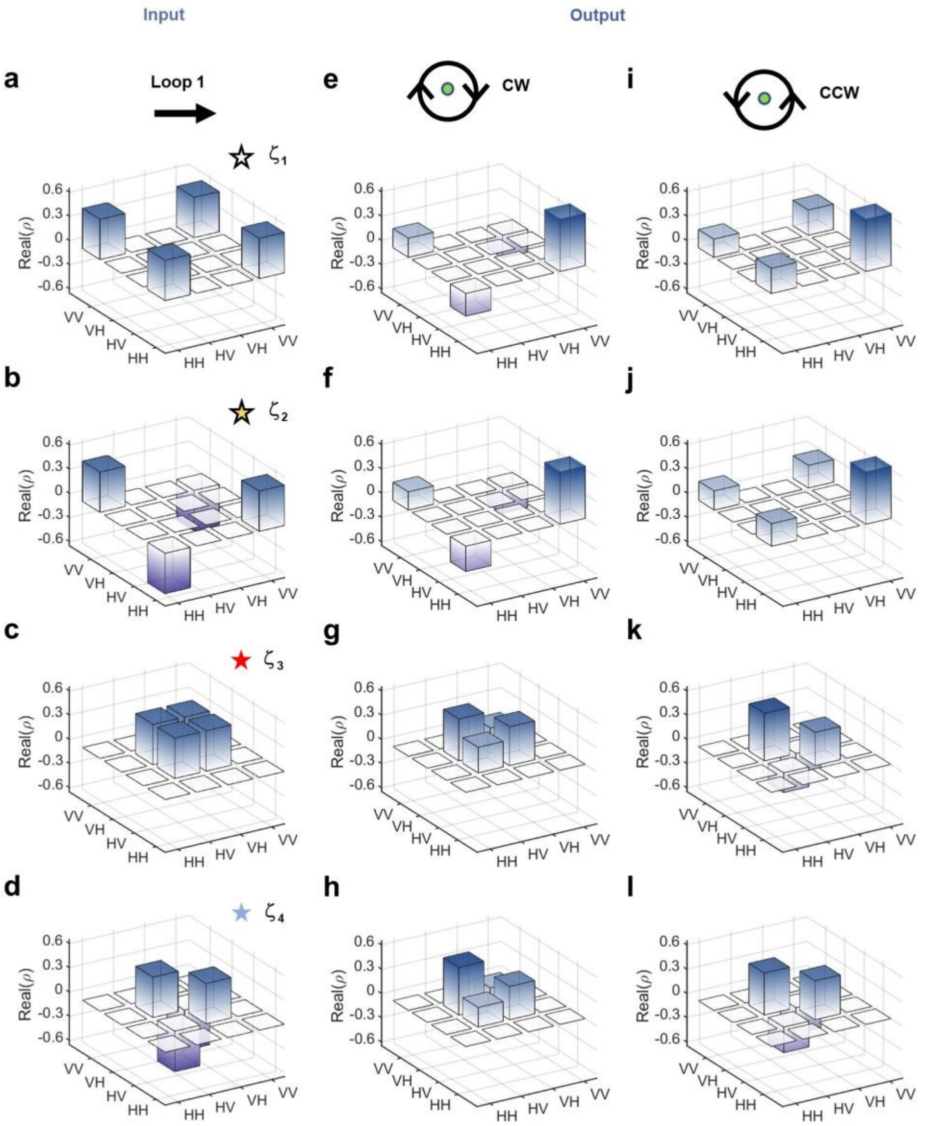

Figure S5. Theoretical results of encircling the EP for $N=8$. a-d Density matrices of the input states $|\zeta_j\rangle (j=1,2,3,4)$. **e-h** Density matrices of the output states after CW encircling, where **e** and **f** show the fidelity between the output states and $|\zeta_2\rangle$, and **g** and **h** show the fidelity between the output states and $|\zeta_3\rangle$. **i-l** Density matrices of the output states after CCW encircling.

We also give the theoretical results of output states for $N=8$, see Fig. S5. For input states

$|\zeta_1\rangle$ and $|\zeta_2\rangle$, encircling the EP clockwise results in output states close to $|\zeta_2\rangle$ (Fig. S5e and S5f), but counter-clockwise results in outputs close to $|\zeta_1\rangle$ (Fig. S5i and S5j). For input states $|\zeta_1\rangle$ and $|\zeta_4\rangle$, the outputs close to $|\zeta_3\rangle$ (Fig. S5g and S5h) are obtained in a clockwise way, while close to $|\zeta_4\rangle$ (Fig. S5k and S5l) in a counter-clockwise way. This demonstrates that encircling the EP enables asymmetric conversion between the four entangled states, i.e., realize a chirality switch for entangled states. The output entangled state in the conversion is determined by the direction of circling the EP, and the conversion efficiency is high. Therefore, in our experiment, we select the parameters θ_1 and φ as in this theoretical discussion.

S4. Experimental results of the imaginary part along Loop1 and demonstration of disappearance of chiral switching behavior along Loop 2

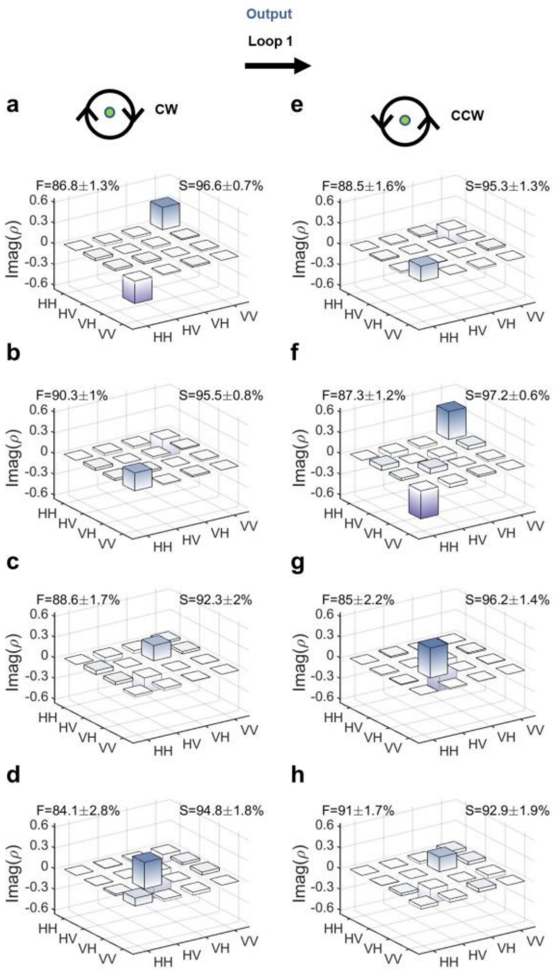

Figure S6. The imaginary part of output density matrix after encircling an EP. a-d Experimental density matrices after clockwise encircling of the EP. **e-h** Experimental density matrices after counter-clockwise encircling of the EP. At the top of **a** to **h**, the symbol F represents the fidelity between the output states and the ideal Bell states. The symbol S represents the similarity $S[\rho_{th}, \rho_{ex}]$ between theoretical and experimental results. From top to bottom, the input states are Bell states $|\zeta_1\rangle$ to $|\zeta_4\rangle$. For all cases, the number of total step is N=8.

In Fig. 4 of the main text, we have provided the real part of output density matrix with different input Bell states. The results show that if the initial states prepared in the experiment are $|\zeta_1\rangle$ and $|\zeta_2\rangle$, the final entangled states obtained are very close to $|\zeta_2\rangle$ when circling the EP clockwise; when circling the EP counterclockwise, the final entangled states obtained experimentally are very close to $|\zeta_2\rangle$. This demonstrates the chiral behavior of the entangled states $|\zeta_1\rangle$ and $|\zeta_2\rangle$ experimentally. In the same system, when the input states in the experiment are $|\zeta_3\rangle$ and $|\zeta_4\rangle$, both change leads to $|\zeta_3\rangle$ with encircling the EP clockwise; while leads to $|\zeta_4\rangle$ with encircling the EP counter-clockwise. Here, in Fig. S6, we supplement the imaginary part of output density matrix. It is found that under the experimental conditions, the imaginary part of output density matrix affects the final results a little. So the output states in our experiment are close to Bell states.

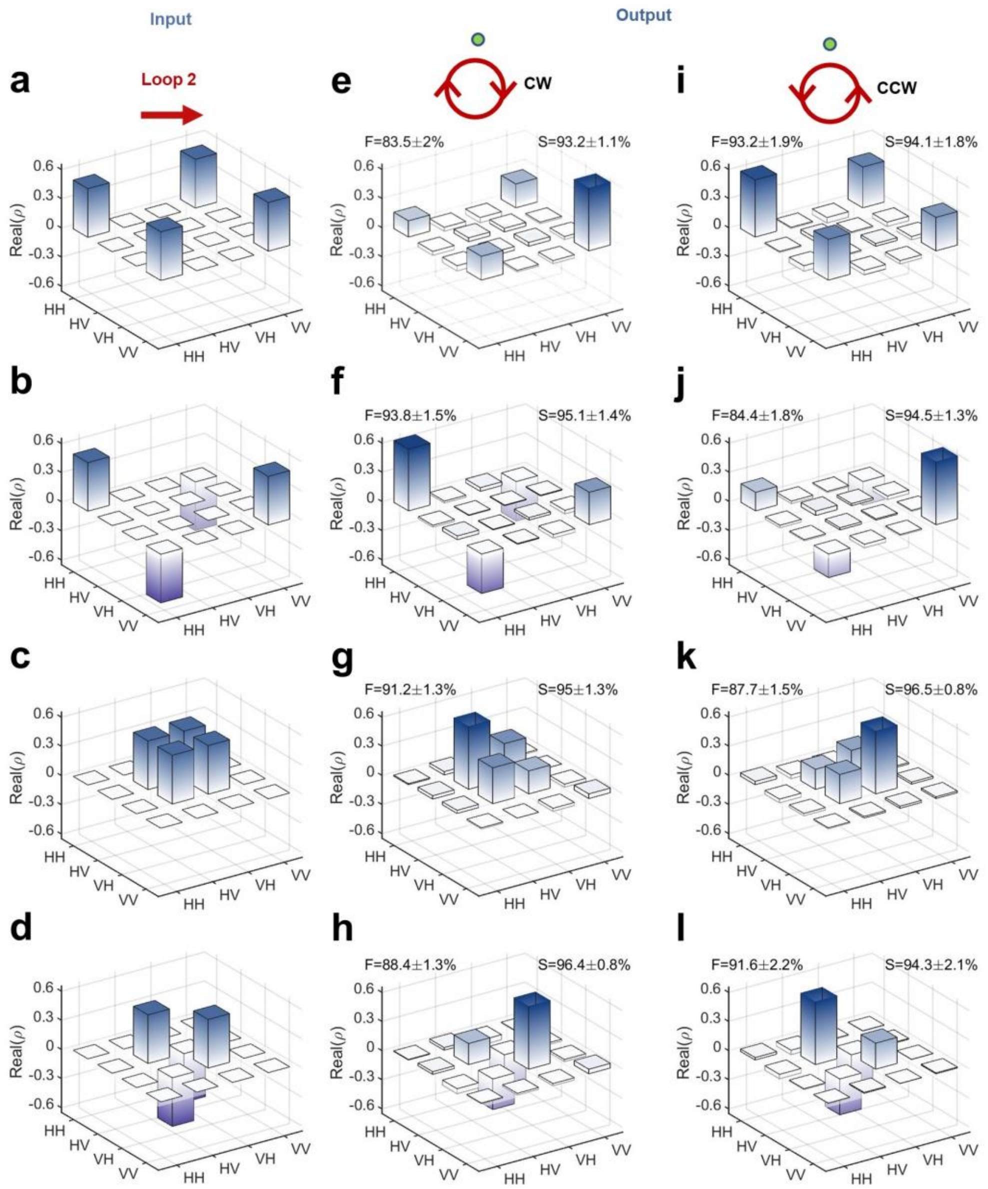

Figure S7. Experimental results along the Loop 2. a-d Density matrices of the four different input Bell states $|\zeta_j\rangle (j=1,2,3,4)$. **e-h** Experimental density matrices after clockwise encircling along Loop 2. **i-l** Experimental density matrices after counter-clockwise encircling along Loop 2. At the top of **e** to **l**, the symbol F represents the fidelity between the output states and the ideal Bell states. The symbol S represents the similarity $S[\rho_{th},\rho_{ex}]$ between theoretical and experimental results.

In the experiment, we also measure the output states for the encircling along Loop 2. As shown in Fig. S7a-S7c, when the initial state is $|\zeta_1\rangle$, the output states are close to the initial

state $|\zeta_1\rangle$, no matter whether encircling the loop clockwise or counter-clockwise. The reason is that the state is not affected by the topological properties of EP during the evolution when the parameters on the loop are far away from the EP. So the output states only display the adiabatic evolution results. The average fidelity of output states between the experiment and theory is larger than 0.95, indicating a good agreement between the experiment and theory. Our results of the Bell state conversion show that the chiral behavior disappears, when the evolution path of parameters is far away from the EP.

S5. The chiral switch of Bell states without encircling the EP.

The above discussions focus on the case for encircling the EPs. Recent investigations have shown that chiral state transfers can appear without encircling the EP. In fact, our designed topologically protected entanglement switching can also work without encircling the EP.

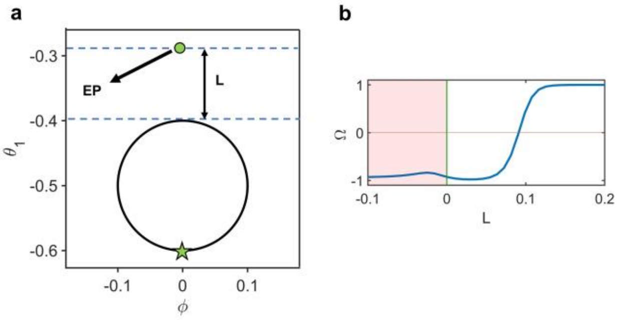

Figure S8. The chiral switch of Bell states without encircling the EP. a The distance L between the EP and loop. **b** The chirality with the change of L.

To reveal the chiral switch quantitatively, we choose the input state as $|\zeta_1\rangle$, and define the relative occupation in the output states as $P = \left(|C_1|^2 - |C_2|^2 - |C_3|^2 - |C_4|^2\right) / \left(|C_1|^2 + |C_2|^2 + |C_3|^2 + |C_4|^2\right)$. The values $|C_{1,2,3,4}|$ represent the coefficients of four Bell states $|\zeta_1\rangle$ to $|\zeta_4\rangle$. So this value P describes the ratio of these four Bell states in the output state. When this value P equal to 1, it means that the output state is just $|\zeta_1\rangle$; while -1 indicates the output state changes to $|\zeta_2\rangle$, $|\zeta_3\rangle$ and $|\zeta_4\rangle$, which is

orthogonal to $|\zeta_1\rangle$. The range of P covers from -1 to 1. We denote P_{CW} and P_{CCW} to represent the ratios with evolving along the loop clockwise and counter-clockwise, respectively. Then provide the quantity chirality as $\Omega = P_{CW} * P_{CCW}$ whose range is also from -1 to 1. The value of $\Omega = 1$ indicates the results for the clockwise and counter-clockwise evolutions are same, which means the lack of chirality for the system. For comparison, the value of $\Omega = -1$ illustrate the existence of asymmetric evolutions between the clockwise and counter-clockwise evolutions.

To explore the effect of distance *L* between the EP and loop, we move the loop along θ_1-axis downwards, and show the change of chirality, see Fig. S8. When *L* is smaller than 0, the loop contains the EP; while when *L* is larger than 0, the loop does not contain the EP. The chirality with *L* has been provided in Fig. S8b. The pink area represents the case with the EP in the loop. The value of Ω is always nearly -1 with the change of *L*, which means the existence of a chiral switch of the Bell state $|\zeta_1\rangle$. When L is larger than 0 a little, the value of Ω is also about -1. This indicates that the chiral switch still exists even the loop does not contain the EP. However, when *L* is far larger than 0, the value of Ω changes from -1 to 1, and keeps 1 with *L* finally. It illustrates the disappearance of a chiral switch of the Bell state $|\zeta_1\rangle$. Similar change behaviors of chirality with *L* can be found for other Bell states, and we do not provide the calculation details here. Based on the discussion above, our designed topologically protected entanglement switching can also work without encircling the EP. The chiral switch of Bell states appears when the loop is close to the EP.
